# Supplementary material for: Interpopulational differences in the nutritional condition of Aequiyoldia eightsii (Protobranchia: Nuculanidae) from the Western Antarctic Peninsula during austral summer
Source: PeerJ. 2021 Dec 21;9:e12679. doi: 10.7717/peerj.12679 (PMC8706337; doi:10.7717/peerj.12679)
Supplement: Supplemental Information 1 — Statistical values are given in the right upper corner, after Student t-test (a) and Mann–Whitney test (b and c). In the boxplot, the horizontal end of the box nearer to zero represents the 25th percentile and the horizontal end of the box more distant from zero represents the 75th percentile. The horizontal black line within the box indicates the median and the red line within the box indicates the mean. Whiskers above and below the box represent 1.5 times the interquartile range from the box, respectively. Black circles above and below the whiskers are outliers n = 178. [file peerj-09-12679-s001.pdf]

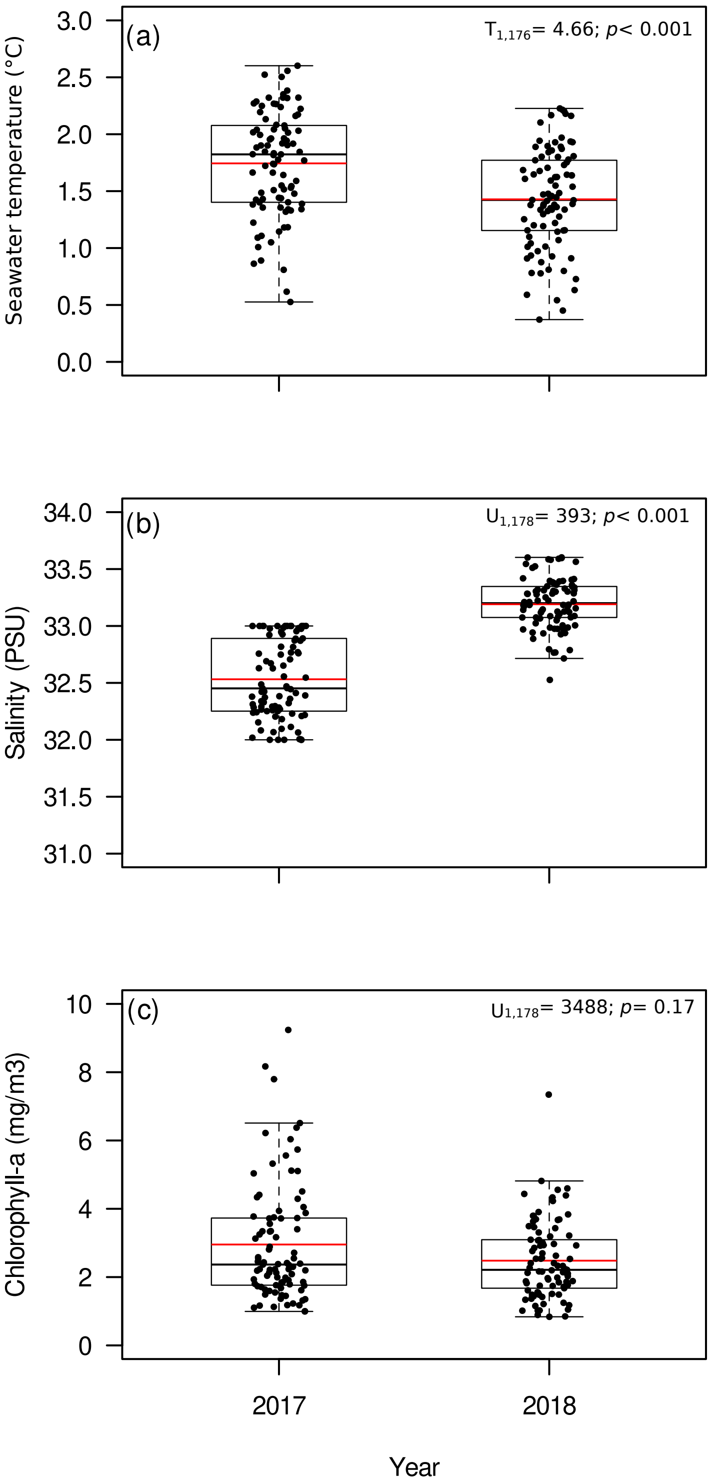

S1 Fig. Jitter boxplot of (a) seawater temperature (ºC), (b) salinity (PSU) and (c) chlorophyll-a (mg/m3) between summer seasons (pooled data January-March) of 2017 and 2018, collected at 0-10m depth at Palmer station (https://pal.lternet.edu/), southern Anvers Island, WAP. Statistical values are given in the right upper corner, after Student t-test (a) and Mann-Whitney test (b and c). In the boxplot, the horizontal end of the box nearer to zero represents the 25th percentile and the horizontal end of the box more distant from zero represents the 75th percentile. The horizontal black line within the box indicates the median and the red line within the box indicates the mean. Whiskers above and below the box represent 1.5 times the interquartile range from the box, respectively. Black circles above and below the whiskers are outliers. n = 178.
