# Supplementary material for: Interpopulational differences in the nutritional condition of Aequiyoldia eightsii (Protobranchia: Nuculanidae) from the Western Antarctic Peninsula during austral summer
Source: PeerJ. 2021 Dec 21;9:e12679. doi: 10.7717/peerj.12679 (PMC8706337; doi:10.7717/peerj.12679)
Supplement: Supplemental Information 2 [file peerj-09-12679-s002.docx]

**Supplemental Table S1. ANOVA table for the shell length of *A. eightsii* individuals collected in three different localities at the WAP.**

| Parameter | Factor | df | MS | F | *p* |
| --- | --- | --- | --- | --- | --- |
| Shell length | Locality | 2 | 11.10 | 2.98 | 0.059^ns^ |
|  | Error | 55 | 3.73 |  |  |
|  | Total | 57 | 14.83 |  |  |

Abbreviations: *df* degrees of freedom, *MS* mean squares, *ns* in the superscript indicates no significant differences.
