# Supplementary material for: Interpopulational differences in the nutritional condition of Aequiyoldia eightsii (Protobranchia: Nuculanidae) from the Western Antarctic Peninsula during austral summer
Source: PeerJ. 2021 Dec 21;9:e12679. doi: 10.7717/peerj.12679 (PMC8706337; doi:10.7717/peerj.12679)
Supplement: Supplemental Information 3 — When significant differences were found, a multiple range test with a Bonferroni correction was used (*p < 0.01; **p < 0.001). [file peerj-09-12679-s003.docx]

**Table S2. Statistical summary of Kruskal-Wallis test for dry mass, biochemical composition and energy content of *A. eightsii* individuals collected in three different localities at the WAP.** When significant differences were found, a multiple range test with a Bonferroni correction was used (**p* < 0.01; ***p* < 0.001).

| Parameter | Locality | *n* | Ranks sum | H | *p* |  |
| --- | --- | --- | --- | --- | --- | --- |
| Body mass (mg) | O’Higgins | 24 | 748.5 | 12.73 | < 0.01* |  |
|  | Yelcho | 19 | 709.5 |  |  |  |
|  | Rothera | 15 | 253 |  |  |  |
| Lipid (mg) | O’Higgins | 24 | 1053.5 | 43.95 | < 0.001** |  |
|  | Yelcho | 19 | 537.5 |  |  |  |
|  | Rothera | 15 | 120 |  |  |  |
| Protein (mg) | O’Higgins | 24 | 1053.5 | 41.93 | < 0.001** |  |
|  | Yelcho | 19 | 537.5 |  |  |  |
|  | Rothera | 15 | 120 |  |  |  |
| Lipid (%DW) | O’Higgins | 24 | 1053.5 | 43.95 | < 0.001** |  |
|  | Yelcho | 19 | 537.5 |  |  |  |
|  | Rothera | 15 | 120 |  |  |  |
| Protein (%DW) | O’Higgins | 24 | 1053.5 | 41.93 | < 0.001** |  |
|  | Yelcho | 19 | 537.5 |  |  |  |
|  | Rothera | 15 | 120 |  |  |  |
| Energy content (mg) | O’Higgins | 24 | 1111 | 49.25 | < 0.001** |  |
|  | Yelcho | 15 | 480 |  |  |  |
|  | Rothera | 19 | 120 |  |  |  |
