# Supplementary material for: Interpopulational differences in the nutritional condition of Aequiyoldia eightsii (Protobranchia: Nuculanidae) from the Western Antarctic Peninsula during austral summer
Source: PeerJ. 2021 Dec 21;9:e12679. doi: 10.7717/peerj.12679 (PMC8706337; doi:10.7717/peerj.12679)
Supplement: Supplemental Information 4 [file peerj-09-12679-s004.docx]

**Supplemental Table S3. PERMANOVA table for the fatty acid composition of *A. eightsii* individuals collected in three different localities at the WAP.**

| Source | df | MS | Pseudo-F | *p*(perm) | Permutations |
| --- | --- | --- | --- | --- | --- |
| Locality | 2 | 2176.5 | 206.68 | < 0.001 | 9953 |
| Residual | 55 | 10.531 |  |  |  |
| Total | 57 |  |  |  |  |

Abbreviations: *df* degrees of freedom, *MS* mean squares.
